# Patient participation in cancer clinical trials: A pilot test of lay navigation

**DOI:** 10.1016/j.conctc.2016.04.005

**Published:** 2016-04-19

**Authors:** Kathleen B. Cartmell, Heather S. Bonilha, Terri Matson, Debbie C. Bryant, Jane Zapka, Tricia A. Bentz, Marvella E. Ford, Chanita Hughes-Halbert, Kit N. Simpson, Anthony J. Alberg

**Affiliations:** a Hollings Cancer Center, Medical University of South Carolina, Charleston, SC, USA; b College of Nursing, Medical University of South Carolina, Charleston, SC, USA; c College of Health Professions, Medical University of South Carolina, Charleston, SC, USA; d Department of Public Health Sciences, Medical University of South Carolina, Charleston, SC, USA; e Department of Psychiatry and Behavioral Sciences, Medical University of South Carolina, Charleston, SC, USA

**Keywords:** Clinical trial enrollment, Clinical trial education, Clinical trial understanding, Barriers to care, Patient navigation, Health disparities

## Abstract

**Background:**

Clinical trials (CT) represent an important treatment option for cancer patients. Unfortunately, patients face challenges to enrolling in CTs, such as logistical barriers, poor CT understanding and complex clinical regimens. Patient navigation is a strategy that may help to improve the delivery of CT education and support services. We examined the feasibility and initial effect of one navigation strategy, use of lay navigators.

**Methods:**

A lay CT navigation intervention was evaluated in a prospective cohort study among 40 lung and esophageal cancer patients. The intervention was delivered by a trained lay navigator who viewed a 17-min CT educational video with each patient, assessed and answered their questions about CT participation and addressed reported barriers to care and trial participation.

**Results:**

During this 12-month pilot project, 85% (95% CI: 72%–93%) of patients eligible for a therapeutic CT consented to participate in the CT navigation intervention. Among navigated patients, CT understanding improved between pre- and post-test (means 3.54 and 4.40, respectively; p-value 0.004), and 95% (95% CI: 82%–98%) of navigated patients consented to participate in a CT. Navigated patients reported being satisfied with patient navigation services and CT participation.

**Conclusions:**

In this formative single-arm pilot project, initial evidence was found for the potential effect of a lay navigation intervention on CT understanding and enrollment. A randomized controlled trial is needed to examine the efficacy of the intervention for improving CT education and enrollment.

## 1 Introduction

In 2015 cancer is expected to cause more than 589,000 deaths in the US [1]. From a societal perspective, clinical trials (CT) are vital to the discovery of efficacious new cancer treatments to reduce the burden of cancer. From the perspective of a cancer patient, CT participation often represents access to clinically advanced treatment options that are delivered with meticulous attention to the treatment protocol. In the US, approximately 20% of cancer patients are eligible for a cancer CT [2]. However only 3–5% of US cancer patients actually participate in a CT [2]. Participation rates are even lower among minority, rural, elderly and other underserved populations [3], [4], [5], [6]. Multi-level factors hinder CT enrollment, including not being offered the opportunity to participate, lack of understanding about CTs, logistical issues such as cost, transportation, complex clinical regimens, as well as systems issues such as lack of physician knowledge about available CTs [7].

To date, most interventions to help patients overcome barriers to CT participation have involved relatively brief educational interventions. However, patient enrollment in CTs has not been impacted by brief interventions such as standard CT educational material [3], [4], [5], [6] and modified consent forms [8], [9]. More tailored and interactive educational interventions have yielded more promising results. In two randomized controlled trials (RCTs) that tested interactive computer-based CT educational formats, interactive formats were associated with greater willingness to enroll in a CT compared to standard CT educational videos or brochures [10], [11]. In two studies that tested CT educational interventions tailored to African Americans [12] or to common patient misperceptions about CTs [11], patients reported greater willingness to participate in CTs following the intervention. Interventions with the greatest potential to increase CT enrollment may be those that are interactive or tailored to patient learning needs; but there are limited data testing these types of interventions.

The National Cancer Institute (NCI) and the American Society of Clinical Oncology (ASCO) recently summarized consensus recommendations for research strategies that need to be tested for improving patient participation in CTs [2]. In this report, patient navigation was recommended as a strategy that warrants testing. In the clinical setting, patient navigation generally refers to strategies that provide personal assistance to help patients overcome specific educational, communication, and logistical barriers to treatment and follow up medical care. The patient navigation approach could be adapted and tested with the goal of overcoming the issues that pose barriers to CT participation. Since patient navigation is an individually tailored and interactive intervention, it has strong potential as a strategy to improve personalized CT decision-making and enrollment.

However, there are many operational definitions of navigation. Applying the concept of navigation in a healthcare setting to improve CT participation, two single arm trials have tested CT nurse navigator interventions, one comprised of African American breast cancer patients and the other of all types of cancer patients [13], [14]. Both studies reported greater CT participation among navigated patients compared to historical controls, providing evidence that patient navigation may be an effective strategy for supporting patients in regard to CT participation [13], [14]. Thus, a lay navigation approach remains to be evaluated. The present study adds to the small but growing evidence base by evaluating a lay navigation intervention designed to provide patients with education about CTs and assistance to overcome barriers to CT participation.

## 2 Methods

A prospective cohort study was conducted to evaluate the feasibility and potential effect of a lay CT navigation intervention on CT understanding and enrollment. The intervention design was guided by the Chronic Care Model [15], [16]. Specifically, for patients who enrolled in the CT navigation intervention, we incorporated CT decision support, linkage to community and health-system resources, education about the CT treatment regimen for patients who chose to participate in a CT to empower them in their role to adhere to the therapeutic regimens, and clinical reminders.

### 2.1 Setting and participants

The study population was comprised of lung and esophageal cancer patients potentially eligible for a therapeutic CT at one of three NCI-affiliated cancer centers in South Carolina and Georgia. To be eligible for this study, patients had to be age 18 or older, planning to receive primary therapy at the cancer center, and be potentially eligible for a therapeutic trial. No limitations were placed on histological type/stage of cancer or cancer recurrence status, as CTs take place across early to late stage cancers. The intervention took place between September 2010 and September 2011. The study was approved by the institutional review boards at each cancer center site, and all participants provided informed consent prior to study participation.

### 2.2 Clinical trial portfolio available at participating study sites

Prior to the start of the study, efforts were made to ensure that trials were available across a wide range of lung and esophageal cancer types and stages. Care was taken to ensure that trial availability reflected the patient population of the representative sites. Across the overall trial portfolio, 47% of trials were single arm trials (14% Phase 1 and 33% Phase 2) and 53% were Phase 3 randomized controlled trials, with similar proportions of single arm and randomized trials by study site. Of the 3 cancer center sites, one site had 19 lung and 3 esophageal trials open, one site had 5 lung and 3 esophageal trials open, and one site had 12 lung and 2 esophageal trials open. All study sites had 1–3 trials open for patients with Stage 1B-Stage 4 non-small cell lung cancer, but only one site had a trial open for stage 1A patients. All study sites had trials open for limited and extensive small cell disease and esophageal cancer.

### 2.3 Intervention design

#### 2.3.1 Choice of lay navigators to deliver the intervention

Lay navigators were chosen to deliver the navigation intervention for several reasons. First, lay navigators who do not have clinical responsibilities can focus solely on addressing non-clinical barriers (sociocultural, economic, organization and individual) that often disrupt CT recruitment and retention. Second, lay navigators can be used as an affordable and sustainable resource to extend the reach of clinical staff in providing patient education and logistic and emotional support. Thus, the non-clinical lay navigators were non-clinical, lay staff who were recruited and trained prior to study implementation. Each study site added one salaried lay navigator who worked 20 h per week for this study. Lay navigator salaries ranged from $30,000–$35,000 per year (prorated to a 20 h week), with educational level ranging from a licensed practical nursing degree to a non-clinical master's degree. At each site, the navigator was supervised by a CT nurse manager and supported by a designated physician champion, whose role was to help ensure the clinical team understood the navigator's support role and responsibilities.

#### 2.3.2 Navigator training

The lay navigators participated in a three-part training program that included a 1.5-day didactic session, shadowing experiences, a 1 day practical session in which role playing was used to reinforce mastery of navigation skills, and bi-weekly conference calls. Details of the training have been previously reported in detail [17], but briefly the training protocol is described below:

For the didactic session, content included: (1) navigator's role and responsibilities to facilitate CT education and provide practical assistance to overcome barriers to care, with a focus on scope of work boundaries (i.e. focus on CT education and support; not on CT enrollment; no clinical advice or counseling), 2) clinical aspects of lung and esophageal cancer, (2) overview of clinical trials, (3) informed consent/confidentiality, with an emphasis on core components of informed consent, neutral presentation of CT option, informed consent as a process, and patient's prerogative to decline a CT or drop out at any time; (4) health literacy, (5) navigation documentation/recordkeeping, and 6) time management/priority setting. To further ensure that all lay navigators had a robust understanding about ethical practices in CT recruitment and their expected roles, each navigator completed the University of Miami's Basic Citi Course Training for Human Subjects Research [18] and the Education Network to Advance Cancer Clinical Trials (ENAACT) Foundation's Training Course on Enhancing Recruitment and Retention Practices among the Medically Underserved [19].

For the shadowing experience, navigators shadowed key clinical team members at their respective sites for a 6-week period who included social workers, financial counselors, patient educators and lung/esophageal nurse coordinators and clinical trials coordinators. Shadowing preceptors used a navigation shadowing check-off sheet to help ensure that each navigator received exposure to the same training content. Learning objectives focused on understanding the role of support personnel, identifying common barriers to care, practicing active listening skills, and practicing referral protocols.

For the 1-day practical training session in role-playing, the entire multi-disciplinary team (physicians, nurses, trial coordinators and navigators) took part in structured lessons in cultural competencies. Video clips of common navigation scenarios were created and used to provide the multi-disciplinary team (including the navigator) with experiential lessons on the process of interacting to resolve patient barriers via a team approach. Objectives focused on the roles of each multidisciplinary team member, barriers to trial enrollment and retention, strategies navigators can use to help patients overcome barriers, strategies required within health system to overcome barriers, and relevant cultural competencies. All participants then took part together in feedback sessions and a performance checklist was used to evaluate the navigator's mastery of navigation skills in cultural competencies. The navigators also received ongoing support by participating in bi-weekly calls with the project team that focused on real-time experiences the navigator's negotiated daily.

#### 2.3.3 Recruitment of patients for CT navigation intervention

Recruitment of patients for the CT navigation intervention was designed to carefully follow the usual process of recruiting patients for a CT. The usual CT recruitment process was that: 1) the study coordinator pre-screened each patient for CT eligibility and informed the provider if the patient was potentially eligible for a CT, 2) the provider further assessed the patient for CT eligibility in the context of the patient's clinical condition and trial portfolio, and 3) for patients who were CT candidates, the provider, accompanied by the clinical team, met with the patient to inform them about their treatment options, including the CT option.

Recruitment of patients for the patient navigation intervention was built upon the process of CT recruitment. First, the study coordinator pre-screened each patient to assess potential CT eligibility and informed the provider if the patient was potentially eligible for a CT. Next, the provider considered potential CT options for the patient and informed the study coordinator if a CT option was going to be presented to the patient. For patients who were going to be presented with a CT option, the study coordinator informed the patient about the CT navigation study and carried out informed consent for the navigation study. The study coordinator carried out the process of informed consent with the patient (rather than the navigator) to avoid the potential for patients to feel obligated to accept the navigator's help. If the patient agreed to participate in the patient navigation intervention, the study coordinator informed the navigator that the patient had accepted navigation services. At that point, the provider, accompanied by the clinical team and newly assigned navigator, met with the patient to present them with treatment options, which included the option to participate in a CT.

#### 2.3.4 CT navigation protocol

For patients who consented to the navigation study, the CT lay navigation intervention started when the navigator first participated in the patient's first appointment with the oncologist and others on the clinical team. Patients were offered a CT option during this first meeting. Information about the purpose of the CT, how the patient fit candidacy for the trial, what trial participation entailed, and patient rights/responsibilities in the trial were discussed.

Following this initial discussion, which included clinicians, the CT lay navigator and patient watched a 17-min educational video developed by the National Cancer Institute together [20]. The lay navigator then discussed the CT with the patient, answered their questions about the CT, or referred the question to the appropriate clinical team member. The lay navigator also screened the patient for potential barriers to care that could affect CT participation using the Patient Navigation Research Program's Barrier Checklist [7]. In some cases, patients completed informed consent to participate in a CT immediately following their visit with the navigator and in other cases they made their decision at a later point, often at the time of their next clinic visit. From this point forward, the lay navigator assisted the patient by 1) communicating reminders to enhance compliance with the clinical trial protocol, 2) being a liaison between the patient and clinical team about the patient's care plan, 3) helping overcome logistical barriers to CT participation, and 4) providing emotional support, either directly or through referrals. Informed consent for CT participation was completed by the CT study coordinator at each site either during this initial encounter or on a return visit, depending on patient preference.

### 2.4 Data sources and measures

Data sources included recruitment and participation logs for the navigation intervention and CTs, interviewer-administered patient surveys and medical records. A research team member administered surveys to the patient prior to CT navigation and after the CT decision to assess CT knowledge. If patients made a CT enrollment decision on the day of CT presentation, the post-CT decision survey was completed that day. If patients made a CT enrollment decision at a follow-up appointment, the post-CT decision survey was completed at the next visit. Patients completed a final survey to assess satisfaction with navigation services and CT participation following treatment completion or withdrawal, whichever occurred first. The measures used in this report are described below.

#### 2.4.1 Socio-demographic and clinical characteristics

Race, ethnicity, gender, marital status, insurance status, and education level were obtained by self-report using items adapted from the Behavioral Risk Factor Surveillance System survey [21]. Age, tumor type, Eastern Cooperative Oncology Group (ECOG) performance status (0 = asymptomatic to 5 = death) and cancer recurrence status (yes, no) were obtained from the medical record. Early stage cancer was defined as stages I/II for non-small cell lung cancer (NSCLC) and esophageal cancer and non-extensive for small cell lung cancer (SCLC). Late stage cancer was defined as stages III/IV for NSCLC and esophageal cancer and extensive for SCLC.

#### 2.4.2 Enrollment and retention outcomes

1) Enrollment rates in the CT lay navigation intervention and 2) CT enrollment and retention rates were recorded on study recruitment and participation logs. Reason codes were recorded for trial-related factors (e.g. performance status, no matching trial), provider factors (e.g. preferred standard of care, lack of time to enroll patient) and patient factors (e.g. no desire for research, transportation issues) for not participating in navigation.

Navigation enrollment was calculated as the percentage of patients who were eligible and consented to be navigated. CT participation among navigated patients was calculated along the trajectory of CT consent, enrollment and retention. Measures included the percent of: 1) navigation enrollees who consented to a CT; 2) navigation consenters who enrolled in a CT and 3) CT enrollees who completed a CT. Because many of the patients in this study had late stage disease, which often requires continual rounds of maintenance therapy to prevent cancer recurrence; CT completion was defined as having completed at least the initial cycle of therapy on a CT protocol (such as initial experimental surgery or initial cycle of chemotherapy).

#### 2.4.3 CT knowledge

The “Seven-Item Knowledge Scale” [22] measured patient understanding of key CT factors such as knowledge of randomization, clinical equipoise, experimental nature of research, and why CTs are conducted. Response options on the scale are True, False and Don't Know, which are transformed to correct vs. incorrect response. Scores for the overall CT Knowledge Scale range from 0 to 7, with 7 being the highest knowledge score. This instrument has been used in previous research studies reported in the literature, but has not undergone formal psychometric evaluation.

#### 2.4.4 Patient satisfaction

The 5-item “Satisfaction with Clinical Trial Participation Scale” [23] measured patient satisfaction with CT participation on a 5-point Likert Scale ranging from Strongly Disagree to Strongly Agree. A 10-item investigator-developed scale measured patient satisfaction with CT navigation using 4-point Likert Scale formats. Agreement with statements about satisfaction with the navigation program was rated on a scale from “Very Dissatisfied to Very Satisfied” and navigation quality on a scale from “Poor to Excellent.”

### 2.5 Data analysis

Data were analyzed in SPSS Version 20. Participant characteristics were summarized using frequency and percent distributions or means (SD). For the CT Knowledge Scale, True, False and Don't Know responses were recoded as correct or incorrect. Don't Know and missing data were categorized as incorrect. The overall scale ranged from 0 to 7, with 7 indicating the highest knowledge. For individual items on the scale, pre-post percent differences in CT knowledge were calculated as: [(post-test percent correct - pre-test percent correct)/pre-test percent correct]. A McNemar Exact Test was used to test the difference in the percent of correct responses at pre and post-test for individual knowledge items. For the overall CT knowledge scale, mean knowledge scores were calculated at both pre and post test by averaging the percentage of correct scores for all participants divided by the number of overall participants. Pre-post differences in mean CT knowledge score were calculated as: [(post-test mean score - pre-test mean score)/pre-test mean score]. To analyze change in overall CT knowledge between pre and post-test, we report statistical significance using both a Paired T-Test and Wilcoxon Signed Rank Test. The Paired T-Test was used as the primary measure of change in overall scale scores because the prior study that used the CT knowledge scale reported their results using the Paired T-Test, and we wanted our results to be comparable with other studies [22]. Additionally the T-test has been demonstrated to be a robust statistical test when assumptions of normality are not met [24]. Two-sided p-values of 0.05 or less were considered to be statistically significant. Satisfaction data were analyzed for individual scale items as percent distributions of Likert Scale values.

Because the purpose of the study was to examine the process of delivering the intervention in medical oncology clinics and to collect preliminary data on the impact of the intervention, a target sample size was not calculated based on formal hypotheses. Sample size was based on the feasibility of recruitment, given the study budget and timeframe for recruitment.

## 3 Results

### 3.1 Navigation intervention recruitment process

**Fig. 1:**
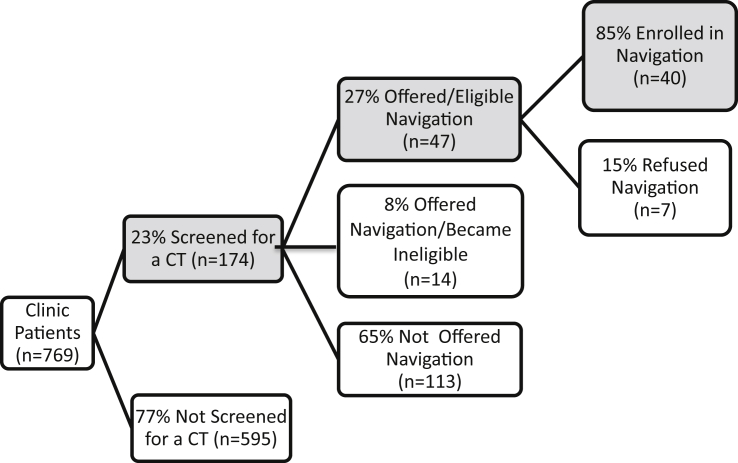
Results for the process of recruitment and enrollment of patients into the clinical trial navigation intervention.

Fig. 1 displays the results for the process of recruitment and enrollment of patients into the clinical trial navigation intervention. During the study period, 769 lung and esophageal cancer patients were identified and of these, 23% (n = 174) were formally screened for a therapeutic CT. Reasons patients were not screened for a CT were due to the lack of a CT matching the patient's cancer (n = 440; 74%), healthcare provider decision (n = 61; 10%), diagnostic work-up indicating no cancer (n = 41; 7%), missed screening opportunity (n = 30; 5%), and other reasons (n = 23; 4%). Only patients formally screened for a CT were eligible for the navigation study.

Among the 174 patients screened for a CT, 27% (n = 47) were offered and eligible to participate in the navigation study, 8% (n = 14) were offered but became ineligible for navigation and 65% (n = 113) were not offered navigation. Reasons patients screened for a CT were not offered or became ineligible for navigation were due to failing CT eligibility or poor health status (n = 39; 64%), provider decision (n = 18; 29%), treatment elsewhere (n = 1; 2%), being missed in clinic (n = 1; 2%), CT refusal (n = 1; 2%) and unknown reasons (n = 1; 2%). Among patients offered and eligible for navigation, 85% (n = 40; 95% CI: 72%–93%) enrolled in navigation. Reasons for navigation refusal were no desire for navigation (n = 3; 43%), feeling overwhelmed by cancer (n = 2; 29%), no desire to participate in research (n = 1; 14%) and passive refusal (n = 1; 14%).

### 3.2 Characteristics of study sample

Table 1 provides the characteristics of the 40 patients who participated in the navigation intervention, along with comparison statistics for those who were offered but did not participate in navigation. The mean age of navigation participants was 63.1 years (SD = 9.56). Seventy three percent of participants (n = 29) were male. Seventy five percent of participants (n = 30) were Caucasian and 25.0% (n = 10) were African American, with only 2.5% (n = 1) reporting Hispanic ethnicity. Ninety three percent (n = 37) had health insurance, 30.8% (n = 13) had a college degree, 67.5% (n = 27) were married or living with a partner, and 66.7% (n = 26) were extremely/quite a bit confident completing medical forms. Eighty five percent of participants (n = 34) had non-small cell lung cancer, 7.5% had small cell lung cancer (n = 3), and 7.5% had esophageal cancer (n = 3). Similarly 85.0% of participants (n = 34) had late stage cancer and 15.0% (n = 5) had cancer of unknown stage. Forty percent of participants (n = 16) received care from the cancer center study site in Charleston (40.0%), 35.0% (n = 14) from Spartanburg, SC and 25.0% (n = 10) from Savannah, GA.

**Table 1:** Characteristics of Navigation Program Enrollees, Those who Agreed to Participate in Navigation but were Unable, and Those who Refused Navigation.

Characteristic	Category a	Participated in navigation study (n = 40)	Agreed to navigation, but unable to participate (n = 14) b	Refused navigation (n = 7)
		N (%)	N (%)	N (%)
Age	Mean (SD)	63.10 (SD = 9.56)	58.86 (SD = 8.99)	68.57 (SD = 7.71)
Race	Caucasian	30 (75.0)	11 (78.6)	7 (100)
	African American	10 (25.0)	3 (21.4)	0 (0.0)
	Other	0 (0.0)	0 (0.0)	0 (0.0)
Ethnicity	Non-Hispanic	39 (97.5)	13 (92.9)	7 (100)
	Hispanic	1 (2.5)	1 (7.1)	0 (0)
Gender	Male	29 (72.5)	10 (71.4)	5 (71.4)
	Female	11 (27.5)	4 (28.6)	2 (28.6)
Insurance Status	Uninsured	3 (7.5)	1 (7.1)	0 (0.0)
	Insured	37 (92.5)	13 (92.9)	7 (100)
Marital Status	Not Married	13 (32.5)	3 (21.4)	3 (42.9)
	Married/living with partner	27 (67.5)	11 (78.6)	4 (57.1)
Cancer Center Study Site	Charleston	16 (40.0)	1 (7.1)	0 (0.0)
	Savannah	10 (25.0)	8 (57.1)	3 (42.9)
	Spartanburg	14 (35.0)	5 (35.7)	4 (57.1)

a Some categories do not sum to 100% due to rounding.

b “Agreed to Navigation, but Unable to Participate” was defined as agreeing to participate in the navigation intervention, but being unable due to declining health or not meeting final eligibility criteria for a clinical trial.

A supplemental analysis was carried out to explore how navigation program participants differed from those who were offered but did not participate in the program. The characteristics of navigation program participants (n = 40) were compared with: 1) those who agreed to navigation, but were unable to participate (n = 14), and, 2) those who refused navigation services (n = 7). Navigation participants were similar to those who agreed to navigation, but were unable to participate in regard to race, ethnicity, gender and insurance status. However navigation participants were older and more likely to be unmarried and from the Charleston study site. In a similar analysis that compared navigation participants with those who refused navigation, these two groups were similar in regard to ethnicity, gender and insurance status. However navigation participants were younger, less likely to be Caucasian and more likely to be married and from the Charleston study site.

### 3.3 CT knowledge outcomes

Among the 35 participants who completed pre and post-test surveys, CT knowledge improved between pre- and post-test (means 3.54 and 4.40, respectively; p-value = 0.004 based on T-Testing) (Table 2). Using a non-parametric testing approach (Wilcoxon Signed Rank Test), a similar p-value of 0.005 was obtained. The greatest knowledge gains were in understanding the concept of randomization and that CTs are not only used when standard treatments have not worked. The item with the lowest percentage of correct baseline responses (n = 5; 14% correct) was for not understanding that their doctor would not know which treatment was better in a CT. No improvement for this item was observed at post-test. For a similar item related to understanding that their doctor would not be able to make sure they got the better treatment in a CT, only 17% of patients (n = 6) answered correctly at pre-test, with 23% answering correctly at post-test (n = 8). This lack of understanding that their doctor is unable to know or ensure that they receive the better treatment in a CT contrasts with the robust improvement from 54% (n = 19) at pre-test to 77% at post-test (n = 27) in understanding that treatment is decided by chance in a CT.

**Table 2:** Comparison between pre-test and post-decision clinical trials knowledge^a^ (n = 35).

Item	Correct at pre-test		Correct at post-test		Post-pre % diff^b^	P-value^c^
	Frequency	Percent	Frequency	Percent	Percent	
In a randomized trial the treatment you get is decided by chance (true)	19	54.3%	27	77.1%	42.0%	0.04
Clinical trials are only used when standard treatment have not worked (false)	17	48.6%	24	68.6%	41.2%	0.04
Clinical trials test treatments which nobody knows anything about (false)	23	65.7%	27	77.1%	17.4%	0.29
Randomized trials are the best way to find out whether one treatment is better than another (true)	27	77.1%	30	85.7%	11.2%	0.45
Clinical trials are not appropriate for serious diseases like cancer (false)	27	77.1%	33	94.3%	22.3%	0.07
My doctor would know which treatment in a clinical trial was better (false)	5	14.3%	5	14.3%	0.0%	1.00
My doctor would make sure I got the better treatment in a clinical trial (false)	6	17.1%	8	22.9%	33.9%	0.63
Mean score (SD) for overall scale	3.54 (1.80)		4.40 (1.14)		24.3%	0.004

a The pre-post CT knowledge analysis includes 35 participants. Of 40 participants enrolled in navigation, 5 were lost to post-test survey follow up due to death (n = 4) and refusal (n = 1).

b For individual scale items, percent difference scores were calculated as (post-test percent/pre-test percent)/pre-test percent. For the overall scale results, percent difference was calculated as (post-test mean/pre-test mean)/pre-test mean).

c A McNamar Exact Test was used to evaluate equality of correct response between pre and post-test for individual knowledge items. A Paired Sample T-Test was used to evaluate the difference between overall mean pre and post test scores. For all analyses, statistical significance was assessed using a 2-sided test at the .05 level.

### 3.4 CT enrollment and retention outcomes

**Fig. 2:**
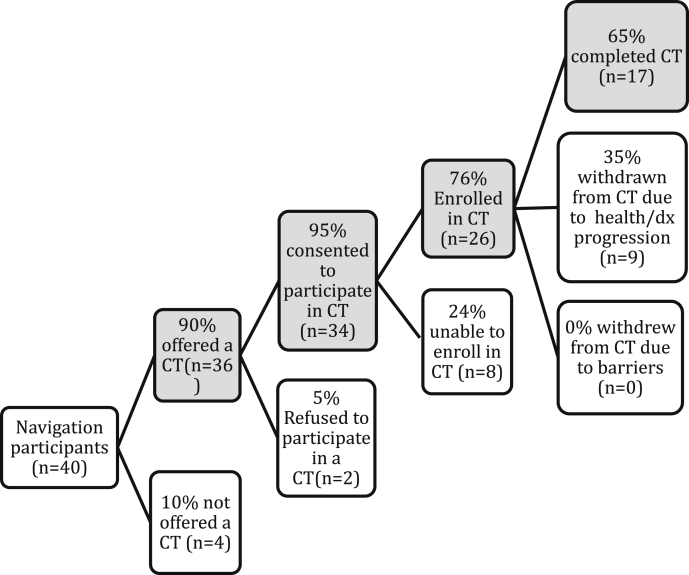
Results for the process of recruitment and enrollment of navigated patients into clinical trials.

Fig. 2 provides CT enrollment and retention results for navigated patients. Among the 40 patients who received CT navigation, 90% (n = 36) were presented with an opportunity to participate in a CT. Reasons patients were not presented with a CT opportunity were due to not meeting final CT eligibility criteria (75%) and death (25%).

Overall, 95% (n = 34; 95% CI: 82%–98%) of patients given an opportunity to participate in a CT did so, 76% (n = 26; 95% CI: 60%–88%) of CT consenters enrolled in a CT and 65% (n = 17; 95% CI: 46%–81%) of CT enrollees completed a CT. Reasons that CT consenters did not enroll in a CT were poor ECOG performance status (0 = asymptomatic to 5 = death) (n = 4; 50%), progressive disease (n = 3; 38%), and abnormal lab values (n = 1; 13%). Thus, because patients were often very sick and CT eligibility requires multi-steps to assess eligibility, it was not uncommon for patients to complete informed consent for a CT, but ultimately not meet final CT eligibility criteria. Reasons that CT enrollees did not complete a CT were drug toxicities/adverse events (n = 4; 44%), disease progression (n = 3; 33%) and death (n = 2; 22%).

### 3.5 Patient satisfaction outcomes

Navigation participants who enrolled in a CT were asked about satisfaction with patient navigation services and CT participation. Among the 24 patients who completed the patient satisfaction survey, all rated the quality of navigation services as good (n = 8; 33%) or excellent (n = 16; 67%) and were satisfied (n = 10; 42%) or very satisfied (n = 14; 58%) with services. All participants agreed (n = 18; 75%) or strongly agreed (n = 6; 25%) they were fully informed about risks and benefits of CT participation. Nearly all participants either agreed (n = 15; 63%) or strongly agreed (n = 7; 29%) that they would recommend CT participation to a friend.

### 3.6 Limitations

Limitations to this study should be considered. First, the vast majority of patients in the three cancer centers did not take part in the navigation study, as only 40 of the 769 cancer center patients participated in the navigation intervention. Thus, the patients included in the navigation intervention do not represent a random sample of clinic patients. The study cohort represents those patients for whom there was a matching CT, whose physician determined that they were appropriate candidates for a CT and who chose to participate in navigation. Second, the study lacked a control group against which to compare intervention results. Third, late stage cancer patients in our study may have been predisposed to participate in research to optimize treatment outcomes. Fourth, there was potential for selection bias if consent to the navigation intervention was correlated with CT consent. Thus, it is not possible to assume causality between the navigation intervention and CT enrollment outcomes. Essentially this study tested a new intervention on a small scale that will need to be further tested in a larger controlled trial.

## 4 Discussion

This study examined the feasibility and signs for a potential effect of a lay navigation intervention to provide cancer patients with CT education and support to overcome barriers to CT participation. The study demonstrated patient willingness to participate in CT navigation, as only 15% of patients refused CT navigation. Patients who refused navigation reported that they did not need help navigating the clinical process. Two CT navigation studies conducted among all types of cancer patients reported navigation program refusal rates of 11% [14] and 44% [25]. Similar to our study, the main reason for CT navigation refusal in these studies was that navigation was not needed. These findings provide evidence that CT navigation is a service that most patients will accept, but that a subset of patients without specific barriers to care will decline. The major limiting factor for enrolling patients in CT navigation was that most patients were not eligible or physically able to participate in a CT. The second most common limiting factor for enrolling patients in CT navigation was physician preference to offer standard of care treatment, suggesting that some patients may have been eligible for a CT, but were not offered the CT navigation intervention. Provider decisions about offering a CT option to their patients are often based on many factors such as knowledge of the available trial portfolio in relation to their patient's condition, time constraints for enrolling and managing the patient on a CT and underlying perceptions about CTs. Thus, it is important to take into account that physician preference for standard of care therapy can be driven by a number of underlying factors.

In this formative study, our recruitment process demonstrated important issues to be addressed in a randomized trial. Based on our study protocol, patients who were formally screened and potentially eligible for a CT were eligible for CT navigation. However, a large proportion of clinic patients were not formally screened for a CT, making them ineligible for the CT navigation intervention. In a future study, systematic screening of all clinic patients for a CT will be paramount to ensuring the optimal recruitment yield within the clinic and to ensuring the generalizability of study findings.

In the present study, CT knowledge increased 24% between pre-navigation and the CT decision. This magnitude of increase is similar to RCTs that focused on increasing knowledge with a CT nurse follow-call (25% knowledge increase) [26] and a total disclosure consent form (24–46% increase) [9], and greater than simply showing patients a CT educational video [27]. Variability in patient understanding of survey items about randomization and the doctor's role in this process were puzzling. Most patients understood that patients are randomly assigned to a treatment in a randomized trial, but did not understand that their doctor is unable to know or ensure they receive the better treatment in a CT. It is possible that this finding may be related to the fact that 47% of trials available in the trial portfolio were not randomized. Patients may have also had a different connotation when they encountered the terms “randomized” vs. “clinical trial” in completing these survey items. It is also possible that patients may have difficulty acknowledging their doctor has no influence in whether they receive the optimal treatment in a CT. Further exploration of the discordance of response across items may help to improve our understanding of how patients process key CT concepts such as randomization. Optimally patients need a complete understanding about their CT option to make an informed CT decision. Thus, a key focus for intervention research will be to ensure alignment of instruments used to measure CT knowledge with specific CT options that patients are being offered and to incorporate a more targeted educational approach that focuses on mastery of a key set of CT knowledge items.

Among patients potentially eligible for a CT, our primary study findings are that 85% (95% CI: 72%–93%) of patients were willing to consent to the CT navigation intervention and of these, 95% (95% CI: 82%–98%) consented to a CT to the extent they were eligible and physically able.

The 95% (95% CI: 82%–98%) CT consent in the current study exceeds the 60% average CT consent reported in observational studies of CT accrual [28], [29], [30]. This finding mirrors results from two CT navigation interventions. In one of these studies conducted in rural American Indians, a CT acceptance rate of 95% was reported [31]. In a nurse navigation intervention that educated and supported cancer patients and providers about available CTs at an academic medical center, 86% of patients accepted CT invitation [13]. When the results of the current study are considered across the continuum of CT consent, enrollment and completion, only one patient declined CT consent. Otherwise, the only reasons that patients failed to consent to, enroll in or complete a CT were due to CT exclusion criteria or health issues that precluded CT participation. These findings provide evidence that structured CT education plus tangible support to communicate with the care team and overcome logistical barriers can help most patients enroll in and complete a CT.

## 5 Conclusions

The results of this pilot study suggest that patient navigation is an intervention with potential to enhance both patient understanding and participation in cancer clinical trials. The study also provides evidence that patients who receive CT navigation services are satisfied with patient navigation and clinical trial services. Based on promising results from this formative pilot work, a randomized controlled trial is needed to examine the efficacy of the intervention on CT understanding and enrollment outcomes.

## Research support

Funding for this study was provided by the National Cancer Institute (P30-CA13831302).

## Previous publications and disclaimers

The results of this study were presented as a poster presentation at the Association of American Cancer Institutes Meeting in Chicago Illinois in July 2011. The authors have no disclaimers to report.

## Conflict of interest statement

The authors declare that there is no conflict of interest.

## References

[1] American Cancer Society (2015). Cancer Facts & Figures.

[2] Denicoff A.M., McCaskill-Stevens W., Grubbs S.S., Bruinooge S.S., Comis R.L., Devine P. (2013). The National Cancer Institute-American Society of Clinical Oncology Cancer Trial Accrual Symposium: summary and recommendations. J. Oncol. Pract./Am. Soc. Clin. Oncol..

[3] Swanson G.M., Ward A.J. (1995). Recruiting minorities into clinical trials: toward a participant-friendly system. J. Natl. Cancer Inst..

[4] Murthy V.H., Krumholz H.M., Gross C.P. (2004). Participation in cancer clinical trials: race-, sex-, and age-based disparities. JAMA J. Am. Med. Assoc..

[5] Du W., Gadgeel S.M., Simon M.S. (2006). Predictors of enrollment in lung cancer clinical trials. Cancer.

[6] Simon M.S., Du W., Flaherty L., Philip P.A., Lorusso P., Miree C. (2004). Factors associated with breast cancer clinical trials participation and enrollment at a large academic medical center. Journal of clinical oncology. Off. J. Am. Soc. Clin. Oncol..

[7] Freund K.M., Battaglia T.A., Calhoun E., Dudley D.J., Fiscella K., Paskett E. (2008). National Cancer Institute Patient Navigation Research Program: methods, protocol, and measures. Cancer.

[8] Coyne C.A., Xu R., Raich P., Plomer K., Dignan M., Wenzel L.B. (2003). Randomized, controlled trial of an easy-to-read informed consent statement for clinical trial participation: a study of the Eastern Cooperative Oncology Group. J. Clin. Oncol. Off. J. Am. Soc. Clin. Oncol..

[9] Simes R.J., Tattersall M.H., Coates A.S., Raghavan D., Solomon H.J., Smartt H. (1986). Randomised comparison of procedures for obtaining informed consent in clinical trials of treatment for cancer. Br. Med. J. Clin. Res. Ed..

[10] Llewellyn-Thomas H.A., Thiel E.C., Sem F.W., Woermke D.E. (1995). Presenting clinical trial information: a comparison of methods. Patient Educ. Couns..

[11] Jacobsen P.B., Wells K.J., Meade C.D., Quinn G.P., Lee J.H., Fulp W.J. (2012). Effects of a brief multimedia psychoeducational intervention on the attitudes and interest of patients with cancer regarding clinical trial participation: a multicenter randomized controlled trial. J. Clin. Oncol. Off. J. Am. Soc. Clin. Oncol..

[12] Banda D.R., Libin A.V., Wang H., Swain S.M. (2012). A pilot study of a culturally targeted video intervention to increase participation of African American patients in cancer clinical trials. Oncol..

[13] Holmes D.R., Major J., Lyonga D.E., Alleyne R.S., Clayton S.M. (2012). Increasing minority patient participation in cancer clinical trials using oncology nurse navigation. Am. J. Surg..

[14] Guadagnolo B.A., Boylan A., Sargent M., Koop D., Brunette D., Kanekar S. (2011). Patient navigation for American Indians undergoing cancer treatment: utilization and impact on care delivery in a regional healthcare center. Cancer.

[15] Wagner E.H., Austin B.T., Von Korff M. (1996). Organizing care for patients with chronic illness. Milbank Q..

[16] Wagner E.H. (1998). Chronic disease management: what will it take to improve care for chronic illness?. Eff. Clin. Pract. ECP.

[17] Bryant D.C., Williamson D., Cartmell K., Jefferson M. (2011). A lay patient navigation training curriculum targeting disparities in cancer clinical trials. J. Natl. Black Nurses Assoc..

[18] University of Miami's Basic Citi Course Training for Human Subjects Research Available: https://www.citiprogram.org/ (accessed 17.03.16).

[19] Education Network to Advance Cancer Clinical Trials (ENAACT) Foundation (2011). Five Steps to Enhance Patient Participation in Cancer Clinical Trials.

[20] NCI (September 2007). Understanding Cancer Clinical Trials (CD/DVD). https://pubs.cancer.gov/ncipl/detail.aspx?prodid=Q021.

[21] Behavioral Risk Factor Surveillance System. CDC website. Available at: http://www.cdc.gov/brfss/index.html.

[22] Ellis P.M., Butow P.N., Tattersall M.H. (2002). Informing breast cancer patients about clinical trials: a randomized clinical trial of an educational booklet. Ann. Oncol. Off. J. Eur. Soc. Med. Oncol./ESMO.

[23] Comis R.L., Miller J.D., Colaizzi D.D., Kimmel L.G. (2009). Physician-related factors involved in patient decisions to enroll onto cancer clinical trials. J. Oncol. Pract./Am. Soc. Clin. Oncol..

[24] Norman G. (2010). Likert scales, levels of measurement and the “laws” of statistics. Adv. Health Sci. Educ..

[25] Steinberg M.L., Fremont A., Khan D.C., Huang D., Knapp H., Karaman D. (2006). Lay patient navigator program implementation for equal access to cancer care and clinical trials: essential steps and initial challenges. Cancer.

[26] Aaronson N.K., Visser-Pol E., Leenhouts G.H., Muller M.J., van der Schot A.C., van Dam F.S. (1996). Telephone-based nursing intervention improves the effectiveness of the informed consent process in cancer clinical trials. J. Clin. Oncol. Off. J. Am. Soc. Clin. Oncol..

[27] Hutchison C., Cowan C., McMahon T., Paul J. (2007). A randomised controlled study of an audiovisual patient information intervention on informed consent and recruitment to cancer clinical trials. Br. J. Cancer.

[28] Lara P.N., Higdon R., Lim N., Kwan K., Tanaka M., Lau D.H. (2001). Prospective evaluation of cancer clinical trial accrual patterns: identifying potential barriers to enrollment. J. Clin. Oncol. Off. J. Am. Soc. Clin. Oncol..

[29] Jenkins V., Fallowfield L. (2000). Reasons for accepting or declining to participate in randomized clinical trials for cancer therapy. Br. J. Cancer.

[30] Klabunde C.N., Springer B.C., Butler B., White M.S., Atkins J. (1999). Factors influencing enrollment in clinical trials for cancer treatment. South. Med. J..

[31] Guadagnolo B.A., Petereit D.G., Helbig P., Koop D., Kussman P., Fox Dunn E. (2009). Involving American Indians and medically underserved rural populations in cancer clinical trials. Clin. Trials.

